# Assessing the effects of a mentoring program on professional identity formation

**DOI:** 10.1186/s12909-023-04748-6

**Published:** 2023-10-25

**Authors:** Lalit Kumar Radha Krishna, Anushka Pisupati, Yun Ting Ong, Kelly Jia Hui Teo, Mac Yu Kai Teo, Vaishnavi Venktaramana, Chrystie Wan Ning Quek, Keith Zi Yuan Chua, Vijayprasanth Raveendran, Harpreet Singh, Sabine Lauren Chyi Hui Wong, Victoria Wen Wei Ng, Eleanor Kei Ying Loh, Ting Ting Yeoh, Jasmine Lerk Juan Owyong, Min Chiam, Eng Koon Ong, Gillian Li Gek Phua, Ruaraidh Hill, Stephen Mason, Simon Yew Kuang Ong

**Affiliations:** 1 Yong Loo Lin School of Medicine, National University of Singapore, 10 Medical Dr, Singapore, Singapore; 2 Division of Supportive and Palliative Care, National Cancer Centre Singapore, Singapore, Singapore; 3 Division of Cancer Education, National Cancer Centre Singapore, Singapore, Singapore; 4 Duke-NUS Medical School, Singapore, Singapore; 5 Lien Centre for Palliative Care, Duke-NUS Medical School, Singapore, Singapore; 6 Health Data Science, University of Liverpool, 200 London Road, Liverpool, UK; 7 Palliative Care Institute Liverpool, Cancer Research Centre, University of Liverpool, 200 London Rd, L3 9TA Liverpool, UK; 8 The Palliative Care Centre for Excellence in Research and Education, PalC C/O Dover Park Hospice, 10 Jalan Tan Tock Seng, Singapore, 308436 Singapore; 9 Centre for Biomedical Ethics, National University of Singapore, Singapore, Singapore; 10 Division of Oncology Pharmacy, National Cancer Centre Singapore, Singapore, Singapore; 11 Assisi Hospice, Singapore, Singapore; 12 Office of Medical Humanities, SingHealth Medicine Academic Clinical Programme, Singapore, Singapore; 13 Division of Medical Oncology, National Cancer Centre Singapore, Singapore, Singapore

**Keywords:** Professional identity formation, Mentoring, Medicine, Professionalism, Palliative medicine, Assessment, Community of practice, Socialisation process, Education environment, Personhood

## Abstract

**Background:**

Medical education has enjoyed mixed fortunes nurturing professional identity formation (PIF), or how medical students think, feel and act as physicians. New data suggests that structured mentoring programs like the Palliative Medicine Initiative (PMI) may offer a means of developing PIF in a consistent manner. To better understand how a well-established structured research mentoring program shapes PIF, a study of the experiences of PMI mentees is proposed.

**Methodology:**

Acknowledging PIF as a sociocultural construct, a Constructivist approach and Relativist lens were adopted for this study. In the absence of an effective tool, the Ring Theory of Personhood (RToP) and Krishna-Pisupati Model (KPM) model were used to direct this dual Systematic Evidence-Based Approach (Dual-SEBA) study in designing, employing and analysing semi-structured interviews with PMI mentees and mentoring diaries. These served to capture changes in PIF over the course of the PMI’s mentoring stages.

Transcripts of the interviews and mentoring diaries were concurrently analysed using content and thematic analysis. Complementary themes and categories identified from the Split Approach were combined using the Jigsaw Approach and subsequently compared with mentoring diaries in the Funnelling Process. The domains created framed the discussion.

**Results:**

A total of 12 mentee interviews and 17 mentoring diaries were analysed, revealing two domains—PMI as a Community of Practice (CoP) and Identity Formation. The domains confirmed the centrality of a structured CoP capable of facilitating longitudinal mentoring support and supporting the Socialisation Process along the mentoring trajectory whilst cultivating personalised and enduring mentoring relationships.

**Conclusion:**

The provision of a consistent mentoring approach and personalised, longitudinal mentoring support guided along the mentoring trajectory by structured mentoring assessments lay the foundations for more effective mentoring programs. The onus must now be on developing assessment tools, such as a KPM-based tool, to guide support and oversight of mentoring relationships.

**Supplementary Information:**

The online version contains supplementary material available at 10.1186/s12909-023-04748-6.

## Introduction

Realising the 2010 Carnegie Foundation’s recommendation [1, 2] to “*explicitly cultivate the formation of professional identity*”, or how medical students “*think, act, and feel like a physician*”, in medical education has met with mixed results [3]. O’Brien and Irby [3] suggest the missing ingredient has been a community of practice (CoP), or “*a persistent, sustaining social network of individuals who share and develop an overlapping knowledge base, set of beliefs, values, history and experiences focused on a common practice and/or enterprise*” [4]. Yet efforts to structure educational programs as CoPs capable of supporting the Socialisation Process—another elemental ingredient in the nurturing of PIF—to facilitate the internalisation of “*the characteristics, values, and norms of the medical profession…, resulting in an individual thinking, acting and feeling like a physician*” have been similarly patchy [5, 6].

Venkataramana et al. [7] suggest that structured mentoring programs may offer a consistent pedagogical solution [8–10]. To these ends, we situate our study of mentee experiences in the Palliative Medicine Initiative (PMI), a structured research mentoring program at the National Cancer Centre Singapore to address our primary research question, “*How do mentees develop PIF in a structured mentoring program?*” and our secondary research question, “*Could a structured mentoring program exhibit features of a community of practice?*”.

### *Palliative Medicine Initiative (PMI)*

The Palliative Medicine Initiative (PMI) is a research mentoring program at the Divisions of Supportive and Palliative Care (DSPC) and Cancer Education (DCE) at the National Cancer Centre Singapore (NCCS) that is open to medical students from Duke-NUS Medical School, National University of Singapore’s (NUS) Yong Loo Lin School of Medicine (YLLSoM), and Lee Kong Chian Medical School. The focus of the PMI is anchored in introducing medical students to research in palliative care, ethics, and professional and medical education. These research projects aim to support mentee first-authored publications in white-listed peer-reviewed journals and/or presentations and posters at national or international conferences.

Since its conception in 2010, the PMI has successfully supported more than 100 single-authored, mentee co-authored and/or mentee first-authored publications in peer-reviewed journals and boasts of more than 150 oral presentations and posters presented at international conferences on palliative medicine, medical ethics, medical education, end-of-life ethics and health services research.

Built on the traditional stages of the research process, the PMI offers a longitudinal, stage-based personalised mentoring approach to medical students [11]. The first stage of the PMI’s mentoring approach begins with a medical student’s agreement to partake in the PMI. Now known as mentees, participating students are first introduced to the different PMI mentors and their projects [10, 12]. Mentees then determine their choice of mentor and project to participate in, having considered the particular goals and timelines of the project. This is then followed by the initial research meeting stage where expectations, codes of conduct, roles and responsibilities are agreed upon by mentees and mentors. This stage also sees the research questions, project goals, timelines and frequency of meetings adapted to the mentee’s abilities, experiences, goals, needs and timelines. Pivotal to this stage is the alignment of project, faculty, and mentee expectations. Subsequently, the stages of data gathering, review of study findings and manuscript preparation expose mentees to the research process whilst nurturing enduring and personalised mentoring relationships [13, 14] that sit at the heart of the PMI’s success [7].

Interactions over the course of the mentoring process are overseen by the host organisation and captured by mentoring diaries [15, 16]. These mentoring diaries are completed by mentees and reviewed by mentors during bi-weekly and ad hoc meetings. A relatively new addition, the mentoring diaries are designed to chart PMI mentees’ development and evaluate their reflective practice [17, 18]. Mentoring diary entries are reviewed by an independent researcher who does not share direct contact or a professional relationship with the mentees in order to safeguard the PMI mentees’ anonymity and privacy. With the mentees’ approval, any psychological, academic, personal, clinical, and/or professional problems or misuse of the mentoring process detected are referred to the psychologist or medical social worker supporting the PMI program. The mentees’ consent is also sought for their anonymised mentoring diaries to be shared and analysed by independent researchers.

### *The PMI as a community of practice*

CoPs share a common identity, approach, values, goals, culture and knowledge requirements within a welcoming environment. To achieve this, CoPs establish clear boundaries that ensure clarity on its remit, membership, structure, trajectory and endpoints. Revealing a structured process, CoPs also render guidance and support mechanisms that facilitate a flexible, personalised adaptable approach to contend with the changing needs of its members. This approach, however, remains firmly within the confines of the program, institutional and professional standards, codes of conduct and expectations.

Notably, CoPs adopt a gradual, stage-based guided immersion into its program—moving members from legitimate peripheral participation at the edges of its boundaries to more central roles within the CoP where they adopt more roles and responsibilities. Guiding this spiralled trajectory is a mix of accessible, timely, holistic and personalised career advice and role modelling, alongside timely feedback, guided reflections, mentoring and peer support. This is made possible by ensuring that the CoP fosters a respectful, mutually supportive and nurturing environment where open discussions and honest feedback can be exchanged and members are challenged to take on new roles and responsibilities.

In light of these features, the PMI can be likened to a CoP for several reasons. To begin, the PMI is supported, overseen, and assessed by the DCE [10, 11]. As the host organisation, DCE seeks to create a sustained network of individuals with a shared “*knowledge base, set of beliefs, values, history and experiences focused on a common practice and/or enterprise*” [4]. To do so, DCE creates a structured mentoring program characterised by clear boundaries, a mentoring trajectory, competency-based mentoring stages that host mentoring assessments, assessment-guided longitudinal mentoring support and a nurturing mentoring environment.

DCE ensures that the PMI has clear boundaries characterised by well-established and consistently applied inclusion criterion; clear codes of conduct and standards of practice; and expectations, timeline, assessment strategies, roles and responsibilities for mentee and peer-mentors that confine practice to a specific area of study [11]. Within these boundaries, DCE maps out the mentoring trajectory for mentees and peer-mentors to achieve first-authored publications in white-listed peer-reviewed journals. This spiral mentoring trajectory that revisits and builds on achieved competencies maps progress from the legitimate peripheral participation to more central roles within the PMI. This spiral trajectory is also scaffolded by clearly defined mentoring stages which, in turn, platform competency-based assessments.

Supporting progress along the mentoring trajectory is the PMI’s adoption of a Combined Novice, Peer and E-mentoring (CNEP mentoring) approach [6, 19] that provides a mix of mentoring, supervision, coaching, role modelling, teaching and instruction (henceforth mentoring umbrella) along the mentoring trajectory [13, 20–24]. This personalised, longitudinal, timely, holistic and appropriate mix of assessment-guided mentoring support fosters and polices mentoring relationships between senior mentors, peer-mentors, host organisation and mentees (henceforth stakeholders) as they progress along the mentoring trajectory and take on graduated responsibilities. It is this personalised blend of support using the mentoring umbrella that allows the PMI to foster the Socialisation Process’s internalisation of “*the characteristics, values, and norms of the medical profession”* [4].

### Mapping professional identity formation

The assumption that the PMI indeed functions like a CoP and supports the Socialisation Process underlines the need to evaluate its ability to nurture PIF. However, an effective means of assessing PIF continues to elude practice, as highlighted by Teo et al. [5]’s review.

**Fig. 1:**
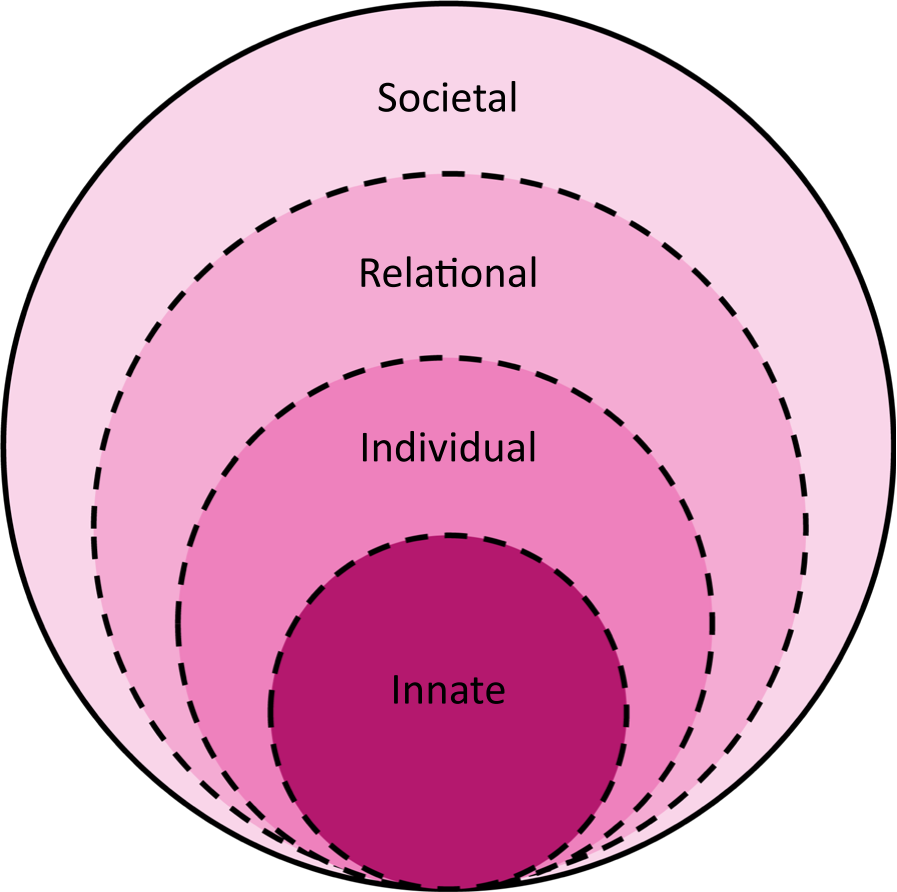
The Ring Theory of Personhood [5]

To this end, we adopt Krishna-Pisupati Model of Professional Identity Formation (henceforth KPM), a theory-backed lens posited to provide an effective sketch of developing PIF in clinical settings [25–27]. Designed on regnant systematic reviews of assessing PIF, the KPM [5] is built around Krishna and Alsuwaigh [28]'s Ring Theory of Personhood (henceforth RToP). The RToP conceptualises personhood as four distinct but related rings, represented by the Innate, Individual, Relational, and the Societal Rings (Fig. 1) [5]. Each ring contains a specific set of beliefs, values and principles (henceforth *belief systems*) that inform a corresponding aspect of a mentee’s identity. The belief system within the Innate Ring revolves around the mentee’s spiritual or religious beliefs, ethical principles and demographic features, such as ethnicity, culture, and gender. The belief system within the Individual Ring captures the mentee’s individuality, autonomous functioning, thoughts, emotions and personality. The Relational Ring is informed by the belief system governing the conduct, standards and expectations surrounding important and personal relationships whilst the Societal Ring guides conduct in the social and professional spheres.

New expectations, practices, insights, reflections, experiences and interactions (henceforth *new life experiences*) change the belief systems within the Innate, Individual, Relational and/or the Societal Rings and with that, different aspects of the mentee’s identity. Building on the reciprocal relationship between belief systems and self-concepts of personhood and identity, the RToP suggests that these changes in belief systems and identity are reflected in the mentee’s self-concept of personhood or ‘*what makes you, you’*. Studies [21, 25, 29–32] have found that when used longitudinally, the RToP can chart changes in a clinician’s personal and professional identity.

The KPM further maps the effects of *new life experiences* on the RToP. Here, the introduction of new experiences and changes to the belief systems inspire reflections and insights. When these insights and reflections echo pre-existing belief systems within the Innate, Individual, Relational and Societal Identities, there is ‘resonance’. ‘Synchrony’ occurs when resonant belief systems are reprioritised to better reflect the existing context and settings. Conversely, when current belief systems are in conflict with *new life experiences*, ‘dissonance’ occurs. Dissonance in one ring is called ‘disharmony’ whilst dissonance between the rings is called ‘dyssynchrony’. The KPM explores the effects of resonance, synchrony, dyssynchrony and disharmony (henceforth event) on the belief system and PIF. Here, the KPM suggests that the detection of an ‘event’ is determined by the mentee’s ‘sensitivity’ whilst their ‘judgment’ displays the significance attributed to the ‘event’. The notion of ‘willingness’ to address the ‘event’ and the mentee’s ability, experience and opportunity to ‘balance’ possible adaptations in response to the ‘event’ culminates in ‘identity work’, or the creation of a context-specific self-concept of identity.

The presence of ‘sensitivity’, ‘judgment’, ‘willingness’, ‘balance’ and ‘identity work’ reflect the Socialisation Process. The Socialisation Process within the KPM is only possible when working within a ‘closed’ or structured mentoring program and a well-surveilled environment. The PMI thus grants an opportunity to study the longitudinal effects of a consistent mentoring approach and the Socialisation Process in a structured research mentoring program.

**Fig. 2:**
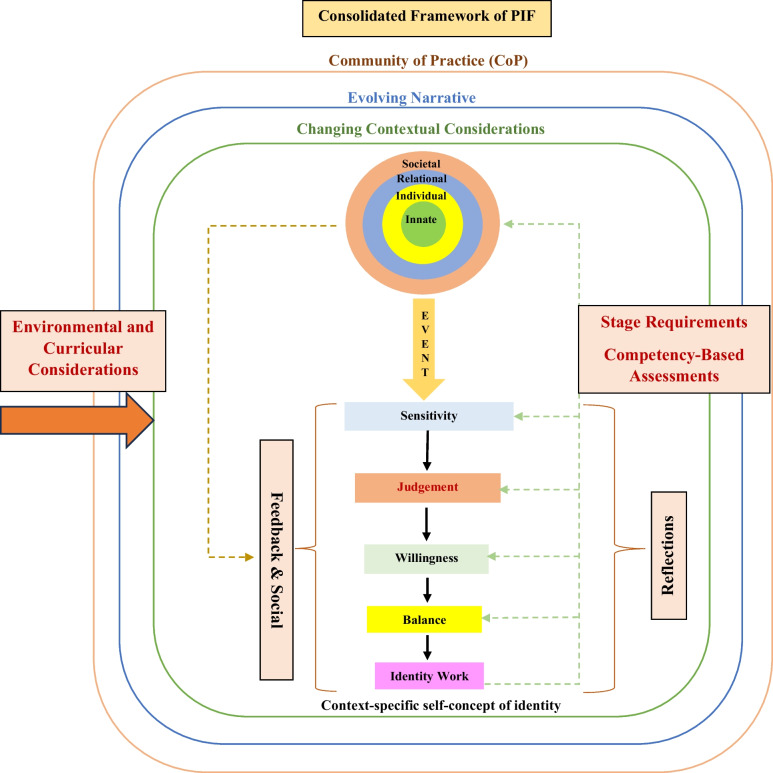
The adapted Krishna-Pisupati Model for professional identity formation [5]

Missing from the current concept of the KPM is an acknowledgement of the effects of the PMI’s use of nonwritten reflections and feedback on the mentee’s narrative, as well the impact of stage-specific expectations, roles and responsibilities on reflections and narratives. Similarly, due consideration of the Socialisation Process and self-concepts of personhood and identity in the face of a mentee’s developing competencies need to be highlighted. Lacking too is an acknowledgement of the effects of environmental factors upon the PMI, including program culture, the wider curricula structure, objectives, goals, settings, sociocultural norms and expectations, legal requirements and the hidden and informal curricula. These findings allow the forwarding of the adapted KPM model (Fig. 2) [5].

### Methodology

To guide our study of PIF amongst mentees in the PMI, we adapted Krishna’s Systematic Evidence-Based Approach (SEBA) [20, 22, 32, 33].

### Theoretical lens

**Fig. 3:**
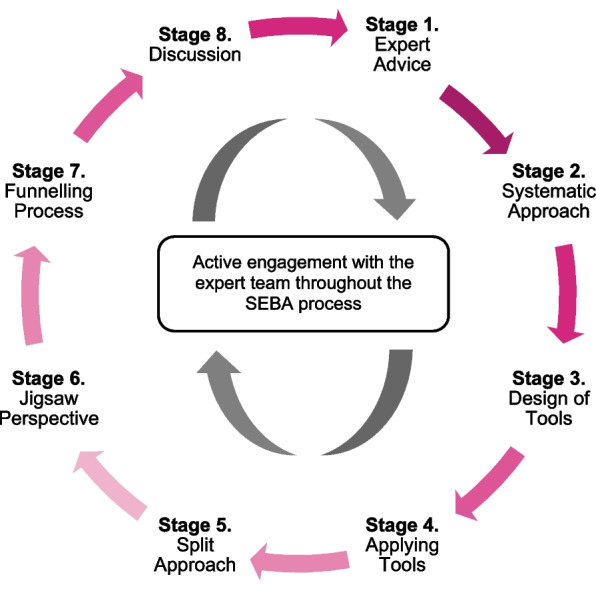
Adapted SEBA process

SEBA’s constructivist approach and relativist lens [5] are best placed to account for PIF as a sociocultural construct [34] shaped by regnant clinical, personal, professional, ethical, psychosocial, cultural and societal factors. The sociocultural nature of PIF is also informed by the mentee’s working styles, motivations, abilities, experience and goals vis-à-vis the mentor and mentee’s individual historical, demographic, socio-cultural, ideological and contextual narratives and the nature, context and duration of their interactions [17, 18]. The stages of SEBA are displayed in Fig. 3.

### Stage 1. Expert team advice

An expert team guided each stage of the SEBA process. The team comprised of a senior female medical librarian with more than 15 years of research experience from the National University of Singapore’s (NUS) Yong Loo Lin School of Medicine (YLLSoM) and local educational experts and clinicians at Duke-NUS Medical School, the National Cancer Centre Singapore, YLLSoM, and the Palliative Care Institute Liverpool. The five male clinicians were all physicians with more than 15 years of clinical, educational and research experience whilst the male educationalists at Duke-NUS Medical School and the Palliative Care Institute held more than 20 years’ experience in medical and clinical research as trained psychologists. All members of the expert team had successfully published at least 5 studies using the SEBA methodology in peer-reviewed journals.

With their primary role of guiding and overseeing the research process, the expert team was not involved in the PMI and thus did not share any contact or prior professional ties with the participants. Concurrently, the research team was tasked with carrying out the research process, data analysis and the writing of the manuscript. Both the expert and research teams were responsible for crafting the research questions.

### Reflexivity

The lead author was a Palliative Care physician and a senior mentor in the PMI with an extensive repertoire of qualitative research experience and peer-reviewed journal publications in medical education and palliative care. As a senior mentor who oversaw the PMI, he was accorded an ‘insider’ role within the study, replete with built-up knowledge and pre-established immersion in the organisation [35]. His in-depth understanding on the mentoring processes and structure within the PMI allowed him to strengthen the rigour of the qualitative study by uncovering nuances and esoteric layers that might not be privy to ‘outsiders’. Access to participants was also smoothened due to the mentor’s proximity to the PMI and its mentees.

However, we also recognised the potential pitfalls that came with being an insider. Prior knowledge on the nuances of the PMI could affect and bias how the lead author viewed, interpreted and analysed the data. Moreover, having a senior mentor of the PMI as the potential interviewer of the study might pose a barrier in encouraging clarifications and unconventional perspectives due to a perceived shared knowledge between the mentor interviewer and mentee participant [36]. The lead author’s valued status as a senior mentor also ran the risk of coercion and social desirability bias [37] in participants where they might conceal their genuine viewpoints to appeal to the mentor. The power imbalance embedded in mentor–mentee relationship was thus a cause for concern in adopting an ‘insider’ role in the study.

In circumventing these roadblocks, we practiced reflexivity by acknowledging and challenging our biases in order to advance trustworthy and honest data. This, for instance, was captured by our recruitment of two trained interviewers who, while part of the PMI, did not share working relationships with the mentees. The absence of a dependent relationship between the interviewers and participants helped mitigate social desirability bias in an ‘even playing field’. Whilst trained on current insights on mentoring and PIF concepts that facilitated effective prompts for robust interviews, the interviewers’ roles as ‘outsiders’ also heightened awareness to unchallenged assumptions otherwise glossed over in shared ‘insider’ relationships [36].

In maintaining reflexivity in data interpretation, the semi-structured interviews were triangulated against anonymised mentoring diaries to enhance data reliability. Furthermore, the lead author consulted members of the expert and research teams comprising of experienced physician-tutors, psychologists, methodologists, and educational scholars to check for any personal biases and attenuate his impact as a senior PMI mentor on the study. The backgrounds of the research and expert teams are detailed in Table 1.

**Table 1:** Expert and research teams demographics

Team Member	Occupation	Qualification	Specialty	Involvement in PMI?
Lead Author	Physician	PhD, MD, MBChB	Palliative Care	Yes
Expert Team Member 1	Psychologist	PhD	Medical Education	No
Expert Team Member 2	Psychologist	PhD	Medical Education	No
Expert Team Member 3	Medical Librarian	PhD	Medical Education	No
Expert Team Member 4	Health Informatics	PhD	Health services research	No
Expert Team Member 5	Pharmacist	Masters (Med Edu)	Medical Education	No
Research Team Member 1	Physician	MBBS	Palliative Care	Yes
Research Team Member 2	Physician	MBBS	Palliative Care	Yes
Research Team Member 3	Physician	MBBS, Masters (Med Edu)	Infectious Diseases	Yes
Research Team Member 4	Physician	MBBS, Masters (Med Edu)	Rheumatology	Yes
Research Team Members 5 to 12	Medical students	Nil	PMI Mentees	No longer actively involved at the commencement of the study
Interviewer 1	Research Manager	Master (Med Hum)	Medical Humanities and education researcher	Yes
Interviewer 2	Research Assistant	Psychology	Clinical Psychology	Hired to do interviews

To ensure input from all members of the team, synchronous and asynchronous in-person and online meetings and Sandelowski and Barroso [3]’s approach to ‘negotiated consensual validation’ were used to reach consensus on the issues discussed.

### Stage 2: Systematic approach

#### Design of the tool

In the absence of an effective tool to evaluate PIF, the expert team advised on the design of semi-structured interview questions drawn from recently published reviews and articles on PIF and mentoring that focused on the PMI framework [10, 11, 20, 38], CNEP mentoring [6, 10, 20, 39], mentoring programs [19, 23, 40–42], mentoring practice [21, 43], mentoring assessments [38, 44–47], the mentoring environment [48] and the influence of the host organisation on the mentoring process [47] published in PubMed, SCOPUS, ERIC, Google Scholar, and Embase databases. This process was guided by the Dual-SEBA guided systematic scoping reviews (SSRs) that had been shown to systematically map and identify key features of PIF [5, 18, 49, 50] and mentoring [6, 7, 20, 39, 49]. Adopting SEBA guided SSRs of PIF and mentoring that complied with the PRISMA guidelines for scoping reviews, the research team used the results to inform the design of the tools.

### Stage 3: Design of interviews and diaries

The combined summaries from Stage 2 guided the design of semi-structured interviews and peer-mentoring diaries around the KPM and RToP [5, 28]. A copy of the interview guide and mentoring diary are enclosed in Additional File 1.

### Stage 4. Applying Tools – conducting interviews and diaries

Recruited through purposive sampling, eligible participants for this study comprised of all PMI mentees in order to best elucidate and capture lived experiences of mentoring and PIF within a structured program. All PMI mentees were sent email invitations by the interviewers which contained the aims of the study, as well as their rights to privacy, anonymity and to withdraw without prejudice.

The semi-structured interviews were conducted over the Zoom video conferencing platform between February and May 2021 by experienced and trained interviewers, AP and CQWL. These 30–45-min online interviews took place in vacant offices that fostered a conducive environment for safe and private in-depth conversations between the interviewers and participants. The two female interviewers did not hold any previous contact with the participants. The participants were informed that both interviewers were experienced in carrying out semi-structured interviews and were employed and trained on current insights on PIF and mentoring for the purposes of this study. Participants consented to having their interviews audio-recorded by a password-encrypted audio-recording device. Using the NVivo 12 Software, all audio recordings were subsequently transcribed verbatim. All transcripts were anonymised.

The peer-mentoring diaries were hosted on Google Forms and were completed between March to December 2021. Anonymisation of mentoring diaries for analysis was expediated by independent research team members not involved the PMI or the semi-structured interviews.

Ethics approval (reference number: 202010–00084 and 202103–00057) was obtained from the Singhealth Combined Institutional Review Board. Informed written and oral consent was obtained from all participants.

#### Stage 5. Split approach

Facilitated by three independent teams of researchers, the Split Approach involved the simultaneous thematic and directed content analysis of the data gathered from the semi-structured interviews and mentoring diaries. The concurrent application of thematic and directed content analysis helped minimise biases, streamline interpretations of terminology by different research team members and address the shortcomings of each method of data analysis. The use of content analysis, for instance, accounted for thematic analysis oft-ignored contradictory data [51–53] and its plausible omission of key categories of interest in existing studies [14, 51–53].

The Split Approach also attended to the neglect of Cohen’s Kappa to assess the degree of consensus between researchers coding the same data [54]. In particular, Kappa inter-reliability scores were not evaluated for this study as the coding process was part of mentor-led training and subjected to frequent expert team input. Sandelowski and Barroso [55]'s approach to ‘negotiated consensual validation’ was thus used to reach consensus on the codes identified.

#### Thematic analysis of interview data

In analysing data collated from the semi-structured interviews, the first team of researchers adopted Braun and Clarke [56]’s approach to thematic analysis that pivoted on coding reliability, the use of code books and a reflexive approach [57]. This involved multiple coders immersed in the data and acknowledgment that their individual viewpoints might influence analysis of the data [58].

To begin, researchers first actively read the anonymised transcripts to discern meaningful patterns in the data [59–63]. Subsequently, they formulated codes derived from the ‘surface’ meaning of the patterns, collating them into a code book for use in an iterative step-by-step analysis process [61]. Each new emerging code was associated with previous codes to form themes that were “defined from the raw data without any predetermined classification” [62]. The research team then discussed their independent findings, adopting ‘negotiated consensual validation’ [55] to finalise the list of themes. Consensus building and use of code books also ensured that assumptions informing the inductive research process were articulated and the results were available for auditing [57].

#### Directed content analysis of interview data

Simultaneously, the second team of researchers utilised Hsieh and Shannon [64]’s approach to directed content analysis to analyse the interview data. This entailed the identification and operationalizing of a priori coding categories from Sarraf-Yazdi et al. [50]’s review entitled, *“A scoping review of professional identity formation in undergraduate medical education”* and Teo et al. [5]’s review entitled, *“Assessing professional identity formation (PIF) amongst medical students in Oncology and Palliative Medicine postings: a SEBA guided scoping review”.* Dubbed the ‘coding agenda’ [65, 66], the codes served a template for deductive content analysis. In effect, concerns surrounding the inconsistency, incoherence and omission of negative results in thematic analysis [9, 25, 31, 50, 58, 67–70] were attenuated. Any data uncaptured by priori codes were assigned new codes [65]. Consensus on the final categories was attained through ‘negotiated consensual validation’ [55, 71].

Notably, Hsieh and Hannon’s approach [64] to directed content analysis also captured all “supporting and un-supporting evidence” on aspects of the RToP within the interview data [64, 66, 71]. The deductive approach adopted facilitated the confirmation and expansion of the RToP theory beyond its traditional role of mapping changes in self-concepts of personhood [66, 72, 73].

#### Analysis of mentoring diary data

Repeating the steps of the two methods detailed above, the third research team carried out thematic and content analysis of the peer-mentoring diaries.

### Stage 6. Jigsaw perspective

Central to the Jigsaw Perspective were Phases 4 to 6 of France et al. [74]'s adaptation of Noblit et al. [75]'s seven phases of meta-ethnographic approach. This approach granted independent researchers the opportunity to reinterpret the data “using a unique synthesis method in order to transcend the findings of individual study accounts” [74]. In comparing the data in a systematic manner, the research team was able to evaluate if the data might be encapsulated within a larger interpretation [74].

Thus, the identified themes and categories were envisioned as pieces of a jigsaw puzzle wherein complementary pieces were merged to paint broader themes. Researchers compared themes and subthemes with the categories and subcategories identified. Similarities were verified through comparisons of the codes contained within them. Should they be complementary in nature, the subtheme and subcategory were combined to create a bigger piece of the jigsaw puzzle.

### Stage 7. Funnelling Process

**Fig. 4:**
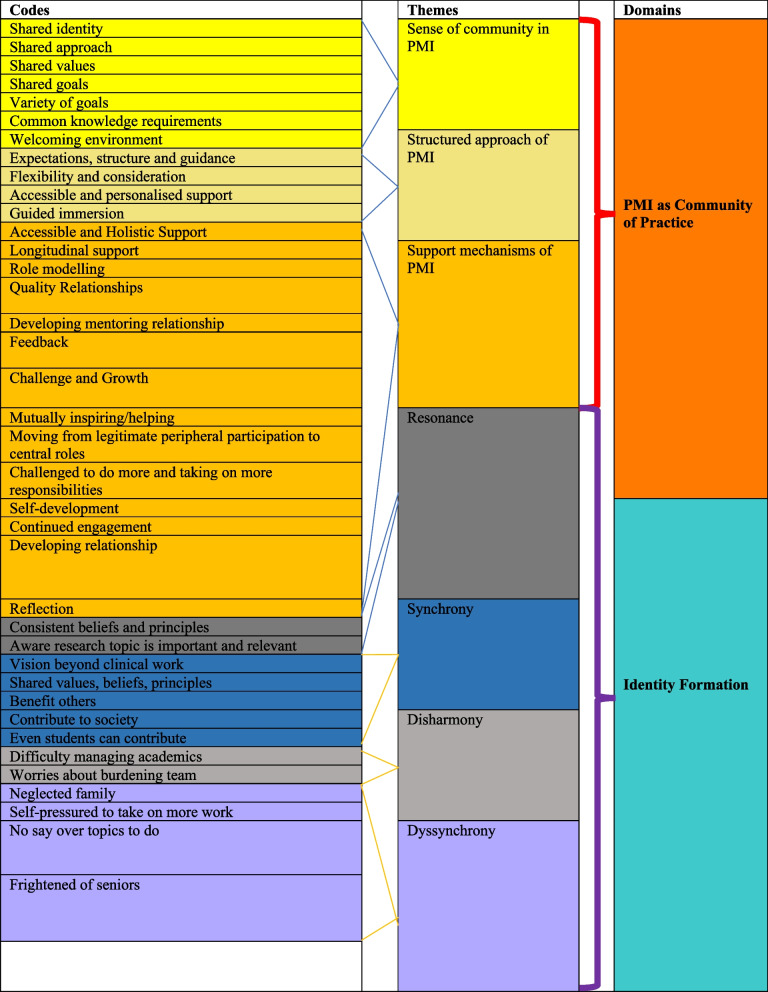
Funnelling Process

The Funneling Process centered on the latter aspects of Noblit et al. [75]'s seven phases of meta-ethnographic approach. Researchers determined if the themes and subthemes were related by systematically comparing and juxtaposing them to form domains. This thus saw the merging of complementary themes from the mentoring diaries and interviews to create domains that framed the discussion in Stage 8 [76] (Fig. 4).

## Results

A total of 20 mentees fulfilled the criteria for participation. Six mentees declined to participate in light of preparations for their examinations. Two mentees did not reply in time. A total of 12 mentees thus participated in the semi-structured audio-recorded interviews while 17 mentees contributed to the mentoring diaries. Table 2 displays demographic information of the participants, including the number of projects that each mentee was involved in to highlight their research experience with the PMI.

**Table 2:** Participant demographics

Study Interviews				
**Mentee**	**Student Year**	**No. of projects**	**Duration involved (years)**	**Peer-Mentor Post-Study**
M1	PGY3	5	4	Yes
M2	Y3	4	2	Yes
M3	Y2	3	1	Yes
M4	Y2	1	1	Yes
M5	Y3	4	1	No
M6	Y4	1	1	No
M7	Y5	4	2	Yes
M8	Y2	1	1	Yes
M9	PGY3	4	5	Yes
M10	Y4	1	2	No
M11	Y4	4	1	Yes
M12	Y3	4	1	No
**Mentoring Diaries**				
**Mentee**	**Student Year**	**No. of projects**	**Duration involved (years)**	**Peer-Mentor Post-Study**
MD1	Y2	2	< 1	No
MD2	Y3	1	< 1	No
MD3	Y1	1	1	No
MD4	Y3	1	< 1	No
MD5	Y2	2	< 1	No
MD6	Y1	2	< 1	Yes
MD7	Y1	2	1	No
MD8	Y1	1	1	No
MD9	Y1	2	1	No
MD10	Y1	1	1	No
MD11	Y3	2	< 1	No
MD12	Y3	2	1	No
MD13	Y2	2	< 1	No
MD14	Y2	3	< 1	Yes
MD15	Y2	1	< 1	No
MD16	Y1	1	< 1	No
MD17	Y3	3	2	No

The interviews and mentoring diaries were evaluated independently. There were no repeat interviews and the concomitant transcripts were member-checked before anonymisation by the interviewers for analysis by the research team.

The two domains identified from Stage 7 were: 1) the PMI as a Community of Practice, and 2) Identity Formation.

### Domain 1. PMI as a Community of Practice (COP)

An initial review of the data indicated that the PMI did display features of a CoP identified in Cruess’ influential paper [77]. Keeping with Clement et al. [78]’s approach and following SEBA’s iterative process, the research and expert teams searched for additional features concurring with CoPs drawn from articles by Sherbino et al. [79], Hean et al. [80], Hägg-Martinell et al. [81], Buckley et al. [82] and de Carvalho-Filho et al. [83] to confirm our initial findings. Addressing the secondary research question, these findings are presented in Tables 3, 4 and 5.

**Table 3:** Sense of Community in the PMI

COP Features	Quotes
Shared Identity	“I think research became a significant part of my medical school identity…it provides some meaning beyond just academics.” (M3)
Shared Approach	“What I really appreciate about the PMI is that everyone is really open to helping other people. They really hold your hand and bring you from the very start, and help you develop from there.” (M12)
Shared Values	“The main values to uphold… is that you have to be open to communicating and responsible for your part of the project.” (M11)
Shared Goal	“Everyone was clear of where we are heading towards, especially after working together for a while.” (MD8)
Variety of Goal	“I joined in order to gain a research experience.” (M11) “It was important for my CV.” (M1)
Common Knowledge Requirements	“[Our near peer mentor] taught us the basic groundwork needed.” (MD11)
Welcoming Environment	“They were really friendly and nice, so there was nothing to be nervous about.” (M10) “It was a community that you could find comfort in… explore and work things out together. So, it was a very supportive system in the sense… we knew that we were not alone.” (M4)

**Table 4:** Structured Approach of the PMI

COP Features	Quotes
Expectations, Structure and Guidance	“They explained ‘what we're trying to achieve, at each stage’, and what was required of me, the deadlines, how to do it and ensured everybody was on the same track.” (M11)
Flexibility and Consideration	“The core team tried to take into consideration our welfare while also ensuring that the various tasks were completed by suitable deadlines.” (M6)
Accessible and Personalised Support	“A lot of guidance along the way… so if like I wrote a manuscript wrongly—he would correct it and I will learn from the new draft that he wrote. This is how I learned; it's really learning on the job.” (M1)
Guided Immersion	“Some of them, I just helped with interviewing, transcribing. Then, I helped to do the write-up. So, as a result of the research process, I've been first author or second author. So, I've done various stages.” (M1) “My mentor was very patient with me and with my peers—to slowly guide us and review the work—to make sure we were doing it right and meeting the standards required. Yeah, he guided us every step along the way.” (M3)

**Table 5:** Support Mechanisms of the PMI

COP Features	Quotes
Accessible and Holistic Support	“I felt afraid to speak about my academic difficulties but… just bit the bullet… And he reassured me that it's okay… And that if I needed to take any time off, it was fine with him.” (M3) “During our mentoring relationship, beyond talking about research, I did look to him for advice in terms of career progression… and he also gave me support in applying for my Master's in medical law and ethics.” (M1)
Longitudinal Support	“If we had any problems in school or like anything in life, we could just talk to him.” (M3)
Role Modelling	“Discipline is definitely something I picked [up] because if I wanted to emulate him in terms of his career progression, I knew that I couldn't remain as lazy as I've always been. So, I did learn to force myself to have some discipline.” (M1)
Quality Relationships	“It has been a great working relationship with all the parties involved.” (MD10) “The people in the project were all quite dedicated to helping each other.” (M12)
Developing Mentoring Relationship	“I'm quite grateful for my mentoring experience… I really learnt a lot in terms of life experience, career guidance, emotional support, life advice.” (M1)
Feedback	“The check-up calls by the mentor were super endearing, candid and offered honest feedback for the improvement.” (MD10) “They also vetted through my papers very thoroughly, even like midway through the writing process.” (M12)
Challenge and Growth	“I think it’s helpful when our mentors do more than just guide us in writing the paper, but challenge us to question why we do certain things.” (MD4) “It’s always nice to discuss different views with others and interesting to see the same papers from a different perspective.” (MD4) “At the start, I tend to step back and follow instructions. Later, I could contribute, and also help the other people… and help the team [to] progress more effectively and progress faster.” (M4) “I was willing to take up my own research project. I was rather happy that my efforts have been acknowledged and that I can get to try out writing a paper myself. From there on, they have been really helpful in guiding us on what we must do.” (MD1) “There was someone who pulled out super last minute… That's where I kind of stepped in… to cover for that person.” (M8)
Mutually Inspiring/Helping	“I've seen him pursue so many things over the years, you know, a lot of passion and a lot of discipline… it did inspire me to pursue my own postgraduate studies.” (M1)
Moving from Legitimate Peripheral Participation to Central Roles	“I started doing the literature review and selecting included articles. Then, thematic analysis and when my senior couldn’t complete the project, I wrote the paper.” (M2)
Challenged to do more and Taking on more Responsibilities	“I just helped with interviewing, transcribing. Then, I helped to do the write-up. So, as a result of the research process, I've been first author or second author. So, I've done various stages.” (M1)
Self-Development	“You do learn how to hold on to your values and the things that matter to you, because other people have their own set of values and morals that you may not necessarily agree with, and there may be conflict because of the different values that you hold. So, I think you just really you learn how to respect that difference and to hold onto the values that matter to you.” (M6)
Continued Engagement	“…it started as a Year 4 student, and he still mentoring me now.” (M1)
Developing Relationship	“…constantly gave us advice on our medical school life and how to cope with our ups and downs in medicine.” (M3)
Reflection	“I definitely get a lot out of it. I think it's difficult to imagine me getting so much out of one mentoring relationship.” (M3)

Table 3 focuses on the PMI creating a sense of community with a shared identity, culture and approach anchored around a variety of common goals and values.

Table 4 highlights the structured approach of the PMI. Here, the guided yet flexible mentoring process outlined clear expectations and rendered personalised support to mentees.

Table 5 foregrounds the support mechanisms of the PMI. These revolved around assessment-driven mentoring support drawn from various approaches within the mentoring umbrella, as well as longitudinal communication and support structures, regular check-ins and feedback sessions, including discussions that extended beyond the research project.

### Domain 2. Identity formation

Based on Venkataramana et al. [7]’s study, PIF may be a function of attending to resonance, synchrony, disharmony and dyssynchrony within the RToP.

#### Resonance

Here, mentees’ regnant values, beliefs and principles were consistent with those of the projects, thus ensuring continued engagement:

“Knowing that our research topic is an important and relevant one and that keeps me going!” (M5)

“This experience made me recognise that the research we are doing isn’t just distant and theoretical, but has important implications in clinical practice as well.” (MD5)

#### Synchrony

Shared values, beliefs and principles empowered mentees to act:

“Shared common values… pass it on… and pay it forward… really resonated with me, especially when an established senior clinician still holds these values and is actively seeking ways to pay it forward… That's the role model that I aspire to be… that's why I joined.” (M9)

“My SM (senior mentor) shared stories about people in his life and showed us why our research was important. At the point, I was starting to feel a little jaded and that meeting was important to remind me the meaning behind our work and gave me a renewed sense of determination to come up with something that would benefit doctors facing the same issue.” (MD1)

“It has shown me how research and its findings can contribute to the current practices within society and that medical students can also play a role in this. I feel like I would be able to make a difference to the medical community and patients if my work is able to get published and perhaps I could be recognised for my work potentially.” (MD17)

“Through our mentoring relationship, I learnt to be a lot more open-minded about things in general, not just in medicine. So, you learn to see things beyond what they are. That's another thing. Resilience because it ties in together with open-mindedness because... partly why I started exploring other non-clinical options for career progression is because I struggled a lot during housemanship.” (M1)

#### Disharmony

Disharmony was reflected in the conflicts that occurred within a single ring of the RToP:

- In the Individual Ring, participants encountered the need to balance personal goals with their own expectations:
  “I didn't do that well for one of my assessments for school and then, I felt that I didn't know if I was capable of taking up so many, too many things at once. And at first, I felt afraid to speak to my mentor about my academic difficulties. Because I was afraid that maybe I wouldn't get opportunities in the future because I cannot juggle my academics with my extra-curricular stuff.” (M3)
  “I feel like I learned that it's very important to dedicate a right amount of time to doing the work because we might feel that, okay, I can finish this within a week. But then, I realised that we shouldn't set such a solid time because you might not know that we actually need more time to go more in detail and find out more about the paper and analyse it to a deeper extent.” (M4)
- In the Societal Ring, conflicts between socially-inspired self-expectations and obligations to the team also emerged:
  “I was very afraid to ask questions and clarify because I was scared that I'll be burdening the team or hampering the effectiveness in the whole efficiency process.” (M3)

#### Dyssynchrony

Dyssynchrony was evidenced in the conflicts experienced between different rings of the RToP:

- Between the Individual and Relational Rings
  “I was neglecting family more… I wouldn't say I'm balancing my time well because I’m putting other things on hold.” (M6)
  “I guess I feel more inclined to take on more work, especially in the smaller team of three. I felt bad because you can't say “I have to take less work because I've other commitments outside” … Because if I don't do it, someone else has to do it and I'm sure everyone is equally busy as well.” (M8)
- Between the Individual and Societal Ring

Here, mentees highlighted the tensions between being in a hierarchical program and their desire to partake in the research process more autonomously or discuss matters outside of research:

“Not having a say over the topics you can get to do or how many papers you get to write… because all of the important critical decisions were made at the top and the information is disseminated downwards.” (M6)

“I was quite scared of seniors in a sense, I didn't really dare to chat seniors up so much and ask things beyond the curriculum.” (M1)

### Stage 8. Discussion

Drawing upon data in Tables 3, 4, and 5, this Dual-SEBA study answers its secondary research question, confirming that the PMI indeed exhibits characteristics of a CoP and supports the Socialisation Process. These findings platform efforts to address the primary research question, “*How do mentees develop PIF in a structured mentoring program?*”*.*

**Fig. 5:**
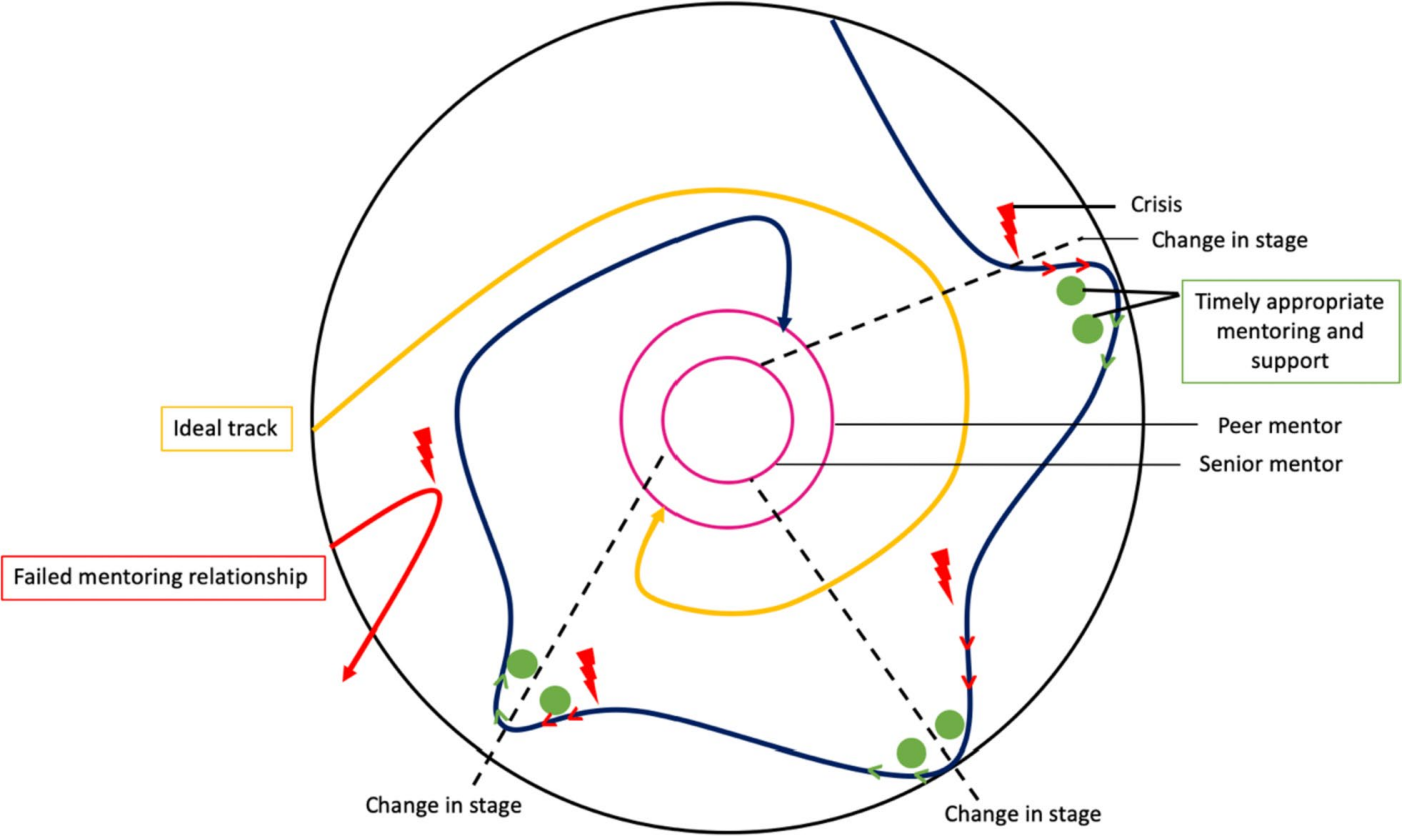
Mentee’s PIF Journey

As a CoP replete with a structured mentoring approach, clear boundaries and competency-based assessments that direct personalised, longitudinal, timely and holistic mentoring support through trained mentors, accessible communication platforms and a curated mentoring environment, the PMI is able to aptly support the Socialisation Process. In turn, the inculcation of the PMI’s shared “*knowledge base, set of beliefs, values, history and experiences*” [4] is made possible through a personalised mix of supervision, coaching, role-modelling, teaching, advising and mentoring support afforded by the mentoring umbrella. Key to the mentoring umbrella’s success are trained mentors and peer-mentors (henceforth faculty). This is further supplemented by the flexibility in adapting a mix of supervised immersion into the mentoring environment, coached competency-building, role-modelling, guided reflections and mentored reflections to meet the mentee’s evolving needs. The constant need to review such support, balance considerations and weigh up the most appropriate response in shifting conditions highlight the importance of longitudinal and personalised assessments by experienced faculty. When applied effectively, the mentoring umbrella helps mentees address resonance, synchrony, disharmony and/or dyssynchrony brought on by new life experiences. This balancing process also considers developing experience, competencies and insights; changing personal and professional circumstances; and access to timely and robust communication and mentoring networks that help steady the rate and magnitude of shifts in the belief system. Such balancing and adaptations are critical as mentees progress from legitimate peripheral participation to more central mentoring roles first within the project and eventually, within the larger PMI program. These results thereupon support and expand upon existing literature on the importance of mentoring in nurturing PIF [84–87]. Notably, these new insights on the development of PIF in the PMI afford the forwarding of a new theory describing the development of PIF and in so doing, answers our primary research question (Fig. 5).

This new theory depicts the PMI as a circle, containing two smaller concentric circles reflecting the graduated progress—first from mentee to peer-mentor and subsequently, from peer-mentor to the mentor’s role at the heart of the PMI. The structure within the CoP is also supplemented by data on the gradual progress from legitimate peripheral participation upon entry into the PMI towards a more central role. This gradual growth is constructed around the PMI’s consistent framework and supported by timely, longitudinal and personalised mentoring support [88]. Successful publications and the adoption of PMI values provide an opportunity for the more mature, experienced and trained mentees to be invited to become a peer-mentor. Progress, however, is not determined by achievement of publication goals or the completion of projects but rather, the development of key competencies, assessment, communication, leadership, mentoring skills and better appreciation of the PMI’s processes.

Upon further training and achievement of these wider skills, knowledge and competencies, ‘senior’ mentees may enter the second ring within the CoP to become peer-mentors. Finally, following a period of mentored oversight of their progress and successful completion of a number of PMI project, alongside achievements of mentoring, leadership, assessment and supportive skills, the peer-mentor may be invited to be a mentor and take their place at the heart of the CoP. It is the mentor and peer-mentor who influence the culture, structure, and environment of the CoP, as well as the mentoring approach and relationships within the PMI. This can be seen from the results in Tables 3, 4 and 5 in which mentors facilitated the adoption of shared values and beliefs, set clear expectations and served as role models for mentees.

Depicting the course through the PMI as a gradual spiral-like process, this proposed theory of PIF in the PMI also aptly discusses the Socialisation Process. Here, we present two spiral trajectories. The size of the spiral represents the speed—a slower progression or rapid adaptation—towards the centre of the PMI. Delineated by the ‘closed’ yellow spiral, this first spiral comprises the ideal PIF trajectory wherein the mentee’s progress occurs uninterrupted towards the centre of the PMI. This is especially so if the mentoring program’s values and practices are synchronous with the mentee’s own identity. This is contrasted against the second spiral in Fig. 5, depicted by the ‘open’ blue spiral that reflects the variability in the course of the PMI journey. This mentee’s experience in the PMI is marked by crises, represented by red lightning bolts in the figure. Each crisis results in dissonance, which sees the mentee’s course ‘veer off’ the desired trajectory [29]. The results from Domain 2 provide a few examples of such crises, including struggling to achieve work-life balance or the tensions within a hierarchical environment. This causes the mentee to fail to achieve deadlines, expectations and competencies, resulting in their trajectories moving away from the centre of the PMI and towards the periphery of the CoP instead. Here, longitudinal assessments, stage-based assessments and regular communications with peers and senior mentors help direct timely, appropriate and personalised support, as depicted by the green dots in the figure. This support aids the mentee in attending to such dissonance and achieve resonance through guided reflections, role modelling, coaching, personalised feedback and remediation provided by peer-mentors and mentors whilst overseen by the host organisation. With effective adaptations to the belief system, deviations in mentee’s trajectory can be redressed and redirected back towards the PMI’s centre.

Moreover, the second spiral highlights two critical considerations. One, the import of longitudinal support, assessment and oversight by trained peer-mentors and mentors. Two, the attenuation of the magnitude of adaptations to the *belief system* due to developing competencies, experiences and insights. Together, this leads to a more ‘stable’ belief system that is less likely to require significant adaptations which, in turn, requires less significant identity work and thus, births more consistent self-concepts of personhood and identity.

The changing nature of these two considerations underscores the importance of longitudinal assessments. With stage-specific mentoring outcomes used as proxy for absent PIF tools, much of it would seem to rest on the shoulders of peer-mentors and mentors. Presently, stage-based mentoring outputs are employed to assess PIF. This underlines the importance of effective selection, mentor and peer-mentor training, matching, longitudinal support and protected time to carry out their mentoring duties. This also highlights the potential for mentoring portfolios that include reflective diaries, stage-based mentoring assessments and reviews by mentors, peer-mentors and peers within the project. Concurrently, the need for adaptive, personalised and timely support of mentees is clear as more robust belief systems and more consistent concepts of professional identity develop. This gradual development of a nuanced context-specific identity and inculcation of PMI values may culminate in an invitation to become a peer-mentor whilst the experiences, insights and growing confidence give mentees a greater sense of ‘belonging’ or identification as part of the PMI, echoing Wenger’s notion of ‘modes of identification’ [89].

These insights help answer our primary research question, “*How do mentees develop PIF in a structured mentoring program?”.* Indeed, this Dual-SEBA guided study underlines the importance of assessment driven, longitudinal, adaptive, individualised and timely mentoring umbrella-based support platformed on an enduring and personalised mentoring relationship. It also underlines that other than the structure and environment of the CoP-like features of the PMI and the role of the curated mentoring environment in nurturing PIF, programs like the PMI rely heavily upon its trained mentors, peer-mentors and its host organisation’s support of them.

### Limitations

This qualitative study has utilised interviews to garner greater insight into the experiences of mentees within the PMI. However, there are some limitations. Whilst it has served to highlight the key elements of PIF, this is first time that the SEBA methodology has been employed in a prospective study, thus requiring further study. Second, whilst the SEBA methodology’s use of independent research and external expert teams helps assuage some issues of bias, continued concerns of bias surrounding some authors’ involvement in the PMI cannot be completely attenuated. Lastly, the use of interviews and mentoring diaries functions as snapshots in time or retrospective recounts which may limit the depth of the data collected.

## Conclusion

A critical feature to be added to this triumvirate of mentoring programs behaving like a CoP supporting the Socialisation Process; the development of enduring and personalised mentoring relationships; and the provision of personalised mentoring umbrella-based support by trained faculty is the need for effective assessments of PIF. As a result, any program in Palliative Medicine and beyond hoping to nurture PIF in a consistent manner requires effective assessment processes built around clear program boundaries; robust and an agreed upon set of expectations and codes of conduct; a well-described mentoring trajectory; aligned expectations; personalised, appropriate and timely application of support akin to the mentoring umbrella; and a nurturing environment. This then will form the focus of our coming research as we look forward to deeper discussions on this increasingly critical area of medical education.

### Supplementary Information

**Additional file 1.**

## Data Availability

All data generated or analysed is included in this article.

## Abbreviations

CoP: Community of Practice
PIF: Professional Identity Formation
RToP: Ring Theory of Personhood
KPM: Krishna-Pisupati Model of Professional Identity Formation
SEBA: Systematic Evidence-Based Approach
