# Supplementary material for: Assessing the effects of a mentoring program on professional identity formation
Source: BMC Med Educ. 2023 Oct 25;23:799. doi: 10.1186/s12909-023-04748-6 (PMC10601320; doi:10.1186/s12909-023-04748-6)
Supplement: Supplementary file 1 — Additional file 1. [file 12909_2023_4748_MOESM1_ESM.docx]

***Additional File 1***

**Interview Guide**

**Demographics**

1. Gender
2. Position/year of study
3. Duration of mentorship: How long have you been mentored?

**DOMAIN 1: RECRUITMENT**

**STAGE 1: PRE-MENTORING**

1. How did you get to know about the program?
2. What attracted you to the program?
3. When enrolling, what was the most important factor?
4. What did you hope to achieve from participating in the mentoring program?
5. What were your expectations of the mentors?
6. Were you aware of what would be expected of you as a mentee when you enrolled?

**Matching**

***Senior Mentor***

1. Did you directly approach your mentor to get involved or were you matched with them?
2. How did you decide on the project topic that you would be working on?
3. If you were matched with your mentor, is there anything you would like to change about the matching process?

***Near Peer Mentor/Peers***

1. Did you select your near peer mentors and/or peers?
2. Were you aware of each other’s roles?

**DOMAIN 2: ALIGNING EXPECTATIONS**

**STAGE 2: INITIAL RESEARCH MEETINGS**

***Initial Meeting***

1. What competencies do you think were expected of you at this stage?
2. Were your roles, responsibilities and timelines explained to you?
3. Was the mentoring approach, reporting system, assessment process and CoPs explained to you?
4. Did you feel the project were in line with your goals/expectations?
5. How did you, your NP and your mentor determine the mentoring approach?
6. What was the agreed upon structure of your specific mentoring process based on your project?

***Trial Period***

1. Was there a trial period agreed upon?
2. Were you aware that you could change your mentor if unsuitable?

**DOMAIN 3: MENTORING PROCESS**

**STAGE 3-5: DATA GATHERING, REVIEW OF INITIAL FINDINGS, MANUSCRIPT PREPARATION**

1. Reflecting on the various stages of the mentoring process, were you told of what was expected of you in each stage of the research process? If not, what competencies do you think were expected of you at these stages?
   1. Did you feel these were reasonable?
   2. Were you guided on this?
   3. What happened if you couldn't meet the goals/expectations?
   4. Did you feel comfortable to ask for help from your NP?
   5. Did you feel comfortable to ask for help from your senior mentor?
2. Were there clear research steps delineated, and how were they determined?
3. How did your NPs and senior mentor check in on you? Eg. email, phone, text
4. How did you know if you were progressing well? Eg. through feedback, clear milestones met?
5. How did the mentor determine if you were progressing well?
6. Did you know that you were being assessed during the mentoring process?
7. Did your mentor tailor their mentoring style when working with you as opposed to using a one-size-fits-all approach?

**DOMAIN 4-5: MENTORING RELATIONSHIP, MENTORING ENVIRONMENT**

1. Did you notice a change in your NP and/or senior mentor’s mentoring style as you progressed?
2. Did the relationship with your NPs and/or senior mentor change along the way? Eg. weaker or stronger, more formal or friendly.
3. Are there any particular incidents that incited such change?
4. How did you address problems:
   1. Within the project
   2. Between you and the senior mentor
   3. Between you and the NP
   4. Between you and other mentees

***Peer***

1. How were you and your peers supported?
2. Did you feel that your relationship with your peers were important to the project?

**DOMAIN 6: NEAR PEER ROLE**

**STAGE 6: REFLECTIONS**

1. Did your senior mentor give you feedback?
2. Did your NP give you feedback?
3. Did you reflect on the given feedback?
4. Have you thought of becoming a NP?
5. When do you feel a mentee might be ready to step up into the role of a NP?
6. What competencies do you think were expected of you at this stage? What competencies should a NP possess?
7. Has your experience in the PMI prepared you to step up into the role of near-peer mentor?
8. Are there shared lessons or reflections gleaned from interactions with your peers?
9. Has mentorship involving your NPs affected your relationship with them in a professional and/or personal capacity?
10. Have there been instances where the roles of mentor and mentee were reversed, where you provided your NP and/or senior mentor with guidance instead?
11. Is there any particular stage of mentoring where you feel you struggled the most in?
12. Did you feel adequately guided?
13. Looking back, do you think you got what you wanted from the program?
14. What were the main positives/negatives you faced?

**PROFESSIONAL IDENTITY FORMATION**

1. Were there experiences, relationships and/or specific beliefs that shaped how you have come to view yourself as a doctor or one soon to be?
2. Please name the most significant aspect that has changed about you.
3. What part of the mentoring process influenced you most?
4. How has your experience in mentoring changed/influenced your
   1. Values
   2. Beliefs
   3. Practice
   4. Thinking- personally, professionally
   5. Attitudes
   6. Self-image
   7. Career goals
5. What is your most enduring memory of the mentoring process?
6. Have your personal, professional, academic and or research circumstances affected the mentoring process? How did you address them?

**Mentoring Diary**

**Mentoring Diaries (Long Ver)**

All information collected will be kept strictly confidential. Thank you!

*Required

Mentee ID *

I grant permission for my ANONYMISED responses to be used for research purposes *

Today's Date *

Date of Last Meeting *

Name of My Near Peer Mentor *

How long have I been involved in the PMI mentoring program (in months/years)? *

How many projects have I been involved in? (in numerics) *

Thinking about the project that I am most involved in, which stage of the mentoring process am I at right now: *

The initial design of the study/project

The search of the databases

Coding

Analysis of the data

Writing the draft

Finalising the manuscript

Submitted the manuscript

Editing the submission based on reviewers comments

Resubmission process or selecting and formatting the article to another journal

Other:

What specific competencies (knowledge, skills and attitudes) are expected of me at this stage? As of today, do I feel confident in these competencies? *

If the response above is ‘no’, why is that so and what steps should I take to resolve this?

How often do I interact with my senior mentor? Does this frequency work for me? *

How often do I interact with my near peer mentor? Does this frequency work for me? *

Thinking about my senior mentor, near peer and fellow mentees, how do I feel the mentoring relationship has been developing? *

|  | Senior mentor | Near peer mentor | Fellow mentees |
| --- | --- | --- | --- |
| Well |  |  |  |
| Satisfactorily |  |  |  |
| Poorly |  |  |  |

Please explain why you feel this way about each of the relationships *

What was a significant mentoring experience I encountered with my senior mentor, near peer and or fellow mentees? (More than one can be described) *

|  | Senior mentor | Near peer mentor | Fellow mentees |
| --- | --- | --- | --- |
| Notable experience |  |  |  |
| What did I learn |  |  |  |
| What feedback would I give to them |  |  |  |

How has my research and mentoring experiences influenced the way I now view

- myself as a medical student/doctor *
- myself within society *
- my relationships with family and friends *
- my general values and beliefs *
- my development of soft skills *
